# Letter to Editor about “Identification of blood-secreted proteins as potential biomarkers for early perinatal depression”

**DOI:** 10.1097/JS9.0000000000003034

**Published:** 2025-07-17

**Authors:** WenLi Zheng, Ying Wang

**Affiliations:** School of Basic Medical Sciences, Zhejiang Chinese Medical University, Hangzhou, Zhejiang, China

*Dear Editor,*

We read with great interest the recent publication by Duan *et al* in the *International Journal of Surgery*, which investigates potential blood-secreted protein biomarkers for early perinatal depression (PPD) using transcriptomic data from the GEO database^[1]^. The study addresses a critical topic in maternal mental health and seeks to uncover molecular markers for early screening. While the research focus is timely and socially relevant, we would like to offer a few critical observations concerning the study’s design, methodology, and interpretation, which may benefit both the authors and future readers.

To begin with, although the study emphasizes the adverse impact of PPD from early pregnancy to the postpartum period and its potential consequences for both mother and child, the discussion on the multifactorial etiology of PPD, such as hormonal fluctuations, psychosocial stressors, and life events, is somewhat limited. Moreover, the limitations of current screening tools (e.g., questionnaires) are not thoroughly addressed, which weakens the case for the urgent clinical need for blood-based biomarkers.

From a methodological perspective, the study relies solely on a single dataset (GSE45603) from the GEO database, comprising a relatively small sample size (53 patients and 13 controls) with insufficient clinical characterization. Critical details such as illness duration, depression severity scores (e.g., EPDS), and history of treatment are missing. This lack of clinical granularity and heterogeneity control may undermine the reliability and reproducibility of the differentially expressed gene analysis. In addition to transcriptomic profiling, exploring DNA methylation patterns such as those available in GSE44132 may offer complementary insights into hub gene regulation and postpartum depression susceptibility^[2]^.

The DEG screening criteria adopted in the study appear relatively lenient, lacking a combined threshold of statistical significance and fold change, which could increase the risk of false positives. Furthermore, integrating multiple analytical approaches, such as Weighted Gene Co-expression Network Analysis, could enhance the robustness of gene discovery. Identifying highly correlated gene modules between the disease and control groups, followed by intersecting them with DEGs, may improve the biological relevance of the findings.

Regarding the results, the authors highlight four secreted proteins, HLA-G, PF4V1, S100A12, and HMGB1, as potential biomarkers. However, no diagnostic model or performance metrics (e.g., ROC curves, AUC values) are provided, and no external validation dataset was employed, limiting the clinical applicability and generalizability of these findings.

Lastly, the study does not account for potential confounders such as socioeconomic status, prior psychiatric history, or postpartum support systems. Important biological variables, including hormonal fluctuations and circadian rhythm disturbances, are also omitted. These factors can significantly influence gene expression and should be considered to strengthen the study’s conclusions.

In summary, the study by Jiang et al. offers preliminary clues toward blood-based markers for perinatal depression. However, there remain notable limitations in study design and data interpretation. We recommend that future research expand the sample size, incorporate multicenter validation cohorts, develop predictive models, and integrate multi-omics approaches to facilitate the development of clinically applicable and translational screening tools.

## Data Availability

Not applicable.

## Research registration unique identifying number (UIN)

There are no conflicts of interest.

## Statement

We wrote according to the TITAN guidelines^[3]^.
